# Identification of *Brucella* RS15060 as a novel type IV secretion system effector associated with bacterial virulence

**DOI:** 10.1186/s13567-024-01417-4

**Published:** 2024-12-18

**Authors:** Yi Yin, Mingxing Tian, Guangdong Zhang, Hai Hu, Chan Ding, Shengqing Yu

**Affiliations:** 1 Shanghai Veterinary Research Institute, Chinese Academy of Agricultural Sciences (CAAS), Shanghai, 200241 China; 2 Shanghai Key Laboratory of Veterinary Biotechnology, School of Agriculture and Biology, Shanghai Jiao Tong University, Shanghai, 200240 China; 3 Veterinary Biopharmaceutical, Jiangsu Key Laboratory for High-Tech Research and Development of Veterinary Biopharmaceuticals, Jiangsu Agri-Animal Husbandry Vocational College, Taizhou, 225300 China

**Keywords:** *Brucella*, label-free quantitative proteomics, T4SS effector, virulence

## Abstract

*Brucella* is an intracellular parasitic pathogen that causes the worldwide zoonotic disease brucellosis. The type IV secretion system (T4SS) is utilized to secrete various effectors to help *Brucella* form *Brucella*-containing vacuoles within the cell and accomplish intracellular trafficking and replication. *Brucella* has fewer recognized effector proteins than other intracellular parasites in the *Proteobacteria*, indicating that *Brucella* may contain a large number of unidentified effector proteins. In this study, the optimal conditions for inducing protein secretion from *Brucella* were screened, and the secreted proteins of 2308 and the T4SS-deficient mutant SV123 under optimal conditions were collected for comparative proteomics analysis. By label-free quantitative proteomics, we identified 15 differential proteins. Through the β-lactamase TEM1 assay and indirect immunofluorescence assay, we identified RS15060 and RS10635 as novel T4SS effectors. Furthermore, by constructing mutation strains and performing cell/mouse infection experiments, we found that deletion of the *rs15060* gene reduced the capacity of *Brucella* to replicate in cells and cause chronic infection in mice. In conclusion, a novel *Brucella* T4SS effector protein, RS15060, was identified to be associated with virulence in this study, and the discovery of effector proteins is conducive to a more comprehensive elucidation of T4SS function as well as to uncovering the cryptic strategies of *Brucella* survival in cells.

**Supplementary Information:**

The online version contains supplementary material available at 10.1186/s13567-024-01417-4.

## Introduction

Brucellosis is a zoonotic disease caused by *Brucella* infection that is prevalent worldwide [1]. Domestic animals, wildlife, and humans are their natural hosts, and infection causes arthritis, osteomyelitis, miscarriages, infertility, and other symptoms [2]. If left untreated, *Brucella* can establish a chronic infection in the host body, and the course of the disease can last for weeks or even years. Brucellosis not only poses a threat to human health but also causes serious economic losses in livestock farming and social production. Understanding the pathogenesis of *Brucella* is essential for preventing and controlling brucellosis.

The key to the pathogenicity of *Brucella* lies in its ability to survive and replicate in host cells. Unlike other pathogenic bacteria, *Brucella* lacks classical virulence factors such as exotoxins, capsules, pilus, and lipopolysaccharides with endotoxin properties [3, 4]. However, *Brucella*-specific factors, such as the type IV secretion system (T4SS), help pathogens adapt to the harsh living environment of host cells, including nutrient deprivation, hypoxia, and acidity, through interactions between effectors and eukaryotic target cells [5], which also induce nutrient synthesis and promote bacterial colonization [5].

T4SS is a multiprotein complex encoded by the *virB* operon [6]. VirB proteins interact with each other to maintain T4SS stability and to facilitate T4SS assembly the at the membrane [6], which aids bacterial secretion of effectors for translocation across the bacterial and host membranes into the host cytoplasm. In addition to the 15 classical T4SS effectors previously reported [7], new effectors have been discovered in recent years. To date, a total of 19 T4SS effectors have been reported to play important roles in mediating *Brucella* intracellular proliferation and manipulating host-related pathways to respond to *Brucella* infection [7–11]. Therefore, on the basis of the size of the genome and the complexity of Brucella pathogenesis, unidentified effectors related to *Brucella* pathogenesis may exist. To identify *Brucella* effectors secreted in a T4SS-dependent manner, we used comparative proteomics analysis to screen for differentially expressed proteins secreted in cultures of *B. abortus* 2308 and its T4SS-deficient strain SV123 in this study. The translocation of the T4SS protein to host cells was subsequently confirmed by a TEM1 reporter assay and indirect immunofluorescence assay (IFA). In addition, protein-deficient strains were constructed to test their role in intracellular replication and the establishment of chronic infection in mice. This study provides novel information on *Brucella* T4SS effectors and *Brucella* pathogenesis.

## Materials and methods

### Bacterial strains, cell culture and growth conditions

*Brucella abortus* strain 2308 and *B. melitensis* strain M5 were obtained from the Chinese Veterinary Culture Collection Center (Beijing, China). *Escherichia coli* strains were routinely cultured aerobically at 37 °C in Luria–Bertani (LB) media (Hopebio, Qingdao, China) or on LB agar plates. The *Brucella* strain and its derivatives were routinely grown in TSB (Difco, Franklin Lakes, NJ, USA) or in this medium supplemented with agar (TSA) at 37 °C under an atmosphere of 5% CO_2_. The following antibiotics were used at the indicated concentrations: kanamycin (Kan; 50 μg/mL), chloramphenicol (Cm; 20 μg/mL), and gentamicin (Gm; 50 μg/mL). The RAW 264.7 macrophage line (ATCC TIB-71, Manassas, VA, USA) was maintained in Dulbecco’s modified Eagle’s medium (DMEM, Gibco, Grand Island, NY, USA) supplemented with 10% (vol/vol) heat-inactivated fetal bovine serum (FBS, Excell, Shanghai, China) at 37 °C in a 5% CO_2_ atmosphere. The composition of the culture medium is described as follows: Gerhardt and Wilson medium [12] contains glycerol, 30 g/L; lactic acid, 5 g/L; glutamic acid, 5 g/L; thiamine, 0.2 mg/L; nicotinic acid, 0.2 mg/L; pantothenic acid, 0.04 mg/L; biotin, 0.0001 mg/L; K_2_HPO_4_, 10 g/L; Na_2_S_2_O_3_·5H_2_O, 0.1 g/L; MgSO_4_, 10 mg/L; MnSO_4_, 0.1 mg/L; FeSO_4_, 0.1 mg/L; and NaCl, 7.5 g/L; adjusted the pH to 6.8–7.0 [13]; Specific Defined Medium contains K_2_HPO_4_·3H_2_O, 9.2 g/L; KH_2_PO_4_, 3 g/L; N_2_S_2_O_3_, 0.1 g/L; NaCl, 5 g/L; nicotinate, 0.2 g/L; thiamine (hydrochloride), 0.2 g/L; pantothenate, 70 mg/L; (NH_4_)_2_SO_4_, 0.5 g/L; MgSO_4_, 10 mg/L; MnSO_4_, 0.1 mg/L; FeSO_4_, 0.1 mg/L; biotine, 0.1 mg/L; and erythritol, 2 g/L; adjusted the pH to 6.8–7.0 [14].

### Plasmids and bacteria

*B. melitensis* M5 is an attenuated strain derived from the virulent *B. melitensis* strain M28 [15]; thus, the primers used were designed on the basis of the whole-genome sequence of *B. melitensis* strain M28 in the NCBI GenBank (RefSeq: NC_017244.1/NC_017245.1). The plasmid pT10 was constructed by amplifying the 200 bp fragment Bcsp31 containing the *bcsp31* promoter from *B. melitensis* M5 via the primers Bcsp31-F/R and connecting it to the linearized pBBR1MCS via the primers Line-pBBR1-F/R. Given that the TEM tag located at the N or C-terminus of the gene may have different impacts on the expression and translocation of the fusion protein, we used both pT10-TEM1-C (*Kpn*I- and *Xba*I-digested) and pT10-TEM1-N (*Xho*I- and *Bam*HI-digested) to construct the fusion plasmids of potential effector-encoding genes amplified from the *B. melitensis* M5 strain. All the inserts were confirmed by sequencing and then introduced into *B. melitensis* M5 by electroporation. Electroporants were selected on TSA plates containing 20 μg/mL chloramphenicol. The expression of these putative effectors was tested by western blot analysis.

The plasmid pT10 was also used to construct vectors expressing 3 × FLAG-tagged C-terminal effector proteins. 3 × FLAG was amplified via an annealing protocol and inserted into the plasmid pT10 (*Kpn*I- and *Bam*HI-digested). The annealing system was as follows: 10 μL of 3 × FLAG-F/R, 10 μL of annealing buffer (NEBuffer™ r3.1, B6003S, New England Biolabs), and the total volume was increased to 50 μL by adding ddH_2_O. The annealing procedure was 95 °C for 2 min, followed by a decrease of 0.1 °C every 8 s until reaching 10 °C. DNA fragments coding for putative effectors were amplified via PCR via the primers X (putative effectors)-3 × FLAG-F/R (Additional file 1). Then, the PCR products were ligated with pT10-3 × FLAG to generate in-frame fusion to the 3 × FLAG epitope and identified via PCR via primers ID-3 × FLAG-F/R and sequenced. Plasmids expressing 3 × FLAG-tagged fusion proteins were introduced into *B. melitensis* M5 by electroporation. The constructs were confirmed by western blot analysis.

Deletion plasmids were constructed using the SacB-assisted suicide vector pKB as described previously [16, 17]. The 5ʹ fragment, which contained the upstream region of the deletion gene, was amplified with primers X (putative effectors)-UF and X-UR, and the 3ʹ fragment, which contained the downstream region of the deletion gene, was amplified with primers X-DF and X-DR. The 5’- and 3’- fragments were fused-amplified using primers X-UF and X-DR and then cloned and inserted into pKB. After identification, the deletion plasmids were introduced into *B. melitensis* M5 by electroporation. Electroporants were selected on TSA plates supplemented with 50 μg/mL kanamycin, and the resulting kanamycin-resistant clones were cultured in TSB and then screened on TSA plates supplemented with 5% sucrose (wt/vol). The deletion mutants were PCR verified with the primers ID-In/Out-X-F/R (Additional file 1), and the clone carrying the right in-frame deletion was named M5ΔX.

The complemented plasmids were constructed using the pMiniTn7TK vector (*Bam*HI- and *Kpn*I-digested) [18, 19] by inserting the full-length deleted fragment and then introduced into the deletion mutants with the helper plasmid [18] via electroporation. Electroporants were selected on TSA plates supplemented with 50 μg/mL kanamycin and confirmed via the primers ID-In/Out-X-F/R (Additional file 1). The complemented strain was named M5ΔX-Com.

All primers used in this study are listed in Additional file 1. The plasmids and bacteria used in this study are listed in Additional file 2.

### RNA extraction and SYBR green qPCR

RNA was extracted using the TRIzol reagent (Invitrogen, Waltham, MA, USA). The bacteria cultured for the indicated times or in different media were collected by centrifugation at 12 000 rpm at 4 °C in 1.5 mL RNase-free EP tubes and then resuspended in 1 mL TRIzol. A Turbo DNA-free kit (Invitrogen) was used to remove DNA contamination.

A total of 2500 ng of RNA was reverse transcribed to cDNA via a PrimeScript RT kit (Takara Bio, Inc., Shiga, Japan) in a total volume of 50 μL, which was performed for 15 min at 37 °C and 5 s at 85 °C. SYBR Green qPCR was performed via 2 × ChamQ Universal SYBR qPCR Master Mix (Vazyme, Nanjing, China) on a Mastercycler ep Realplex system instrument (Eppendorf AG, Hamburg, Germany) with the following procedure: 30 s at 95 °C; 40 cycles at 95 °C for 10 s and 60 °C for 30 s; and a melting curve acquisition program. For each gene, qPCR was performed in triplicate, and the relative transcript levels were determined using the 2^−ΔΔCt^ method. *B. abortus gapdh* was used as an internal control for data normalization. All primers used for qPCR are listed in Additional file 1.

### Western blot analysis

Bacterial lysates were resolved via sodium dodecyl sulfate‒polyacrylamide gel electrophoresis (SDS‒PAGE) and then transferred onto nitrocellulose (NC) membranes (Schleicher & Schuell GmbH, Dassel, Germany) at 240 mA for 90 min in an ice bath. Five percent nonfat milk (Sangon Biotech, Shanghai, China) in TBST (TBS-0.05% Tween 20) was used as a blocking buffer for a 1.5 h incubation at room temperature to attenuate the nonspecific binding of the antibody to the membrane. After three washes with TBST, the membrane was probed with a primary antibody (rabbit anti-β-lactamase) (Abcam, Cambridge, UK) at 4 °C for 16 h and a secondary antibody (HRP-conjugated anti-rabbit polyclonal antibody) (Thermo Fisher Scientific, Waltham, MA, USA) for 1 h at room temperature. Imaging was developed via an enhanced chemiluminescence (ECL) detection reagent kit (Share-bio, Shanghai, China), and the samples were scanned via a chemiluminescence scanner (Tanon-5200).

### Proteomics

*B. abortus* 2308 and the *virB1-3*-deficient mutant 2308∆*virB123* (abbreviated as SV123) were cultured in TSB until OD_600_ = 2.0 and centrifuged at 8000 rpm for 10 min at 4 °C. The bacterial sediment was washed twice with RPMI 1640 and then resuspended in 1 L of pH = 4.5 RPMI 1640, followed by induction in a shaker (Crystal Technology Industries, Inc. Dallas, Texas, USA) at 150 rpm for 4 h at 37 °C. After centrifugation at 8000 rpm for 10 min at 4 °C, the resulting supernatant was filtered to remove bacterial residues through vacuum system filters (Corning, CLS431098) and confirmed to be sterile by culturing 100 μL of the filtrate on a TSA plate. The filtrate was then ultrafiltered at 5000 × *g* for 50 min at 4 °C until the filtrate was concentrated to a volume of 1–2 mL. The protein in the filtrate was precipitated using a fourfold volume of anhydrous ethanol at 4 °C overnight. After centrifugation at 8000 rpm, the sediment was resuspended in 300 μL of SDT lysis buffer (4% SDS, 100 mM Tris–HCl, and 1 mM DTT, pH 7.6) in a new 1.5 mL EP tube.

The amount of protein was quantified with a BCA protein assay kit (Beyotime, Shanghai, China). UA buffer (8 M urea, 150 mM Tris–HCl, pH 8.0) was used to remove the detergent, DTT, and other low-molecular-weight components via repeated ultrafiltration. The reduced cysteine residues were blocked by adding 100 μL of iodoacetamide (100 mM IAA in UA buffer) and incubated for 30 min in the dark. The filter was washed three times with 100 μL of UA buffer and then twice with 100 μL of 25 mM NH_4_HCO_3_ buffer. Finally, the filtrate containing the digested peptides was collected by resuspension in 40 μL of 25 mM NH_4_HCO_3_ buffer containing 4 μg of trypsin (Promega) and incubated overnight at 37 °C. The filtrate was desalted on a C18 cartridge (Empore™ SPE Cartridges C18 [standard density], bed I.D. 7 mm, volume 3 mL, Sigma), concentrated by vacuum centrifugation and reconstituted in 40 μL of 0.1% (v/v) formic acid. Each sample was separated via an easy nLC (Thermo Fisher Scientific), an HPLC liquid phase system with a nanoliter flow rate. After chromatographic separation, the samples were analysed by mass spectrometry using a Q-Exactive mass spectrometer (Thermo Fisher Scientific). MaxQuant software was used for library identification and protein quantitative analysis.

Bioinformatics analysis was performed on the data analysis platform APPLIED PROTEIN TECHNOLOGY called APT-BioCloud [20].

### TEM1 protein translocation assay

The translocation of translational fusions of TEM1 and *Brucella* candidate proteins in infected RAW 264.7 cells was assessed by detection of β-lactamase activity. The plasmids identified by PCR and sequencing were electroporated into the *B. melitensis* M5 strain to construct the TEM1 secretory activity reporter strain X (putative secretory protein)-TEM1, which has a TEM1 tag at the gene’s C-terminus, and TEM1-X, which has a TEM1 tag at the gene’s N-terminus. The expression of the TEM1 fusion protein was verified by western blot analysis with an anti-β-lactamase antibody as the primary antibody (Abcam, Cambridge, UK). RAW 264.7 cells were plated in 96-well plates (tissue culture-treated, black-wall, clear-bottom plates, Corning 3603) at 6.25 × 10^4^ cells per well and incubated for 24 h. *B. melitensis* M5 and its derivatives were cultured to the mid-log phase, and their OD_600_ was adjusted to 1.0. RAW 264.7 cells were infected with *Brucella* at a multiplicity of infection (MOI) = 1000:1 (bacteria:cells) by centrifugation at 400 × *g* for 5 min and incubated for 1 h at 37 °C in 5% CO_2_, which was recorded as 0 hour post-infection (hpi). After the cells were washed three times with prewarmed DMEM, fresh DMEM containing 50 μg/mL gentamicin was used to kill the extracellular bacteria and maintain cell growth. The cells were washed with Hanks’ buffer at 5 hpi, and 6 × CCF2-AM substrate loading solution was prepared according to the protocol of the LiveBLAzer™ FRET-B/G Loading Kit (Thermo Fisher Scientific). A volume of 20 μL of 6 × substrate loading solution was added to the wells containing 100 μL of cells in Hanks buffer or the cell-free background control wells. Wells with glutathione S-transferase (GST) expression were used as negative controls, and wells with BPE123-TEM1 or TEM1-VceC expression were used as positive controls [21, 22]. The plate was covered with light and incubated at room temperature for 90 min.

In the absence of β-lactamase reporter activity, the fluorescent CCF2 substrate molecule remains intact, and fluorescein emits a green fluorescence signal with an emission peak at 520 nm. When β-lactamase reporter activity is present, a blue fluorescence signal with an emission peak at 447 nm is produced. In a population of cells loaded with the CCF2 substrate, blue fluorescence has β-lactamase reporter activity, whereas cells that appear green do not. Wells without cells or with GST-expressing cells were used as the background control and negative control, respectively. The fluorescence values of the wells were read at 447 nm and 520 nm by a plate reader (Bio-Tek, USA) and calculated as follows: (1) calculate the average emission for the background control for the blue (447 nm) and green (520 nm) channels to obtain the average blue background and average green background; (2) subtract the average blue background from all negative controls and sample blue emissions; and (3) subtract the average green background from all negative controls and sample green emissions to obtain the net blue signal and net green signal for each well. (4) The ratio of blue to green fluorescence was calculated by dividing the net blue signal by the net green signal for each well. (5) The average blue-to-green ratio for the negative controls was determined to obtain the average negative ratio. (6) The response ratio of the samples was calculated as the blue-to-green ratio of the samples/blue-to-green ratio of the average negative ratio. According to the formula for the response ratio, all the data were normalized so that the negative control wells had a mean value of 1.0; thus, a sample was considered positive when its response ratio was significantly different from that of the negative control wells.

### Cell infection

RAW 264.7 cell monolayers in a 24-well cell plate were washed twice with prewarmed DMEM and then infected with *Brucella* strains at an MOI of 200:1 via centrifugation at 400 × for 5 min, followed by a 1 h incubation at 37 °C. The infected cells were washed three times with prewarmed DMEM to remove bacteria that had not adhered to the cells and then incubated for another 1 h after treatment with 100 μg/mL gentamicin to kill residual extracellular *Brucella*. Following three washes with prewarmed DMEM, 1 mL/well of DMEM containing 1% FBS and 20 μg/mL gentamicin was added to maintain cell growth. Two hundred microliters of 0.2% Triton X-100 aqueous solution was added to each well at the indicated times, and CFUs were counted to evaluate the intracellular survival of *Brucella* strains.

### Indirect immunofluorescence assay

RAW 264.7 cells were plated on glass coverslips in 24-well culture plates and infected as described above. At 5 hpi, the coverslips were washed with PBS, fixed overnight in 4% (w/v) paraformaldehyde, and then processed for immunofluorescence labelling of effector proteins with a monoclonal anti-FLAG M2 antibody (1:1000, F1804, Sigma‒Aldrich, USA) as the primary antibody and Alexa Fluor 555-conjugated goat anti-mouse IgG (1:1000 dilution, Thermo Fisher Scientific, Waltham, MA, USA) as the secondary antibody. The coverslips were incubated with rabbit anti-*Brucella* polyclonal antibody (1:1000 dilution, prepared in our lab) and Alexa Fluor 488-conjugated goat anti-rabbit IgG (1:1000 dilution, Thermo Fisher Scientific, Waltham, MA, USA) to track the intracellular bacteria. Representative confocal micrographs were acquired under a Nikon microscope (DS-Ri1, Japan).

### Mouse infection

Female BALB/c mice, aged six to eight weeks, were purchased from Shanghai JieSiJie Laboratory Animal Co., Ltd. (Shanghai, China). Intraperitoneally, five mice per group were infected with 1 × 10^6^ CFU of *Brucella* M5 or its variants. Spleen samples were obtained aseptically at 28 days post infection, weighed, and homogenized in 3 mL of PBS. To measure the number of bacterial CFUs, the tissue samples were diluted in a tenfold series, and 100 μL was subsequently plated on TSA plates for CFU counting.

### Ethics statement

The animal experiment was conducted under a protocol approved by the Institutional Animal Care and Use Committee at Shanghai Veterinary Research Institute, the Chinese Academy of Agricultural Sciences, in strict accordance with the guidelines in the Guide for the Care and Use of Laboratory Animals (Permission#: SV-20230319-06). On the basis of our experience with previous infection models, we used the minimum number of animals required to obtain reproducible findings. In a biosafety level 3 facility, BALB/c mice were housed in microisolator cages with unlimited access to food and water. An intraperitoneal injection of 50 µg/g body weight sodium pentobarbital was used to anaesthetize the mice. The mice were anaesthetized before being cervically dislocated to end their lives.

### Statistical analysis

Statistical analysis was performed via GraphPad Prism 8 (GraphPad, USA) software. Student’s *t* test was used to determine the statistical significance between two experimental groups. One-way ANOVA was used to determine the statistical significance of three or more groups of data. Two-way ANOVA was used to determine the statistical significance of the data between two groups. A *P* value < 0.05 indicated a statistically significant difference.

## Results

### Optimal conditions for inducing secretory proteins in *Brucella*

**Figure 1:**
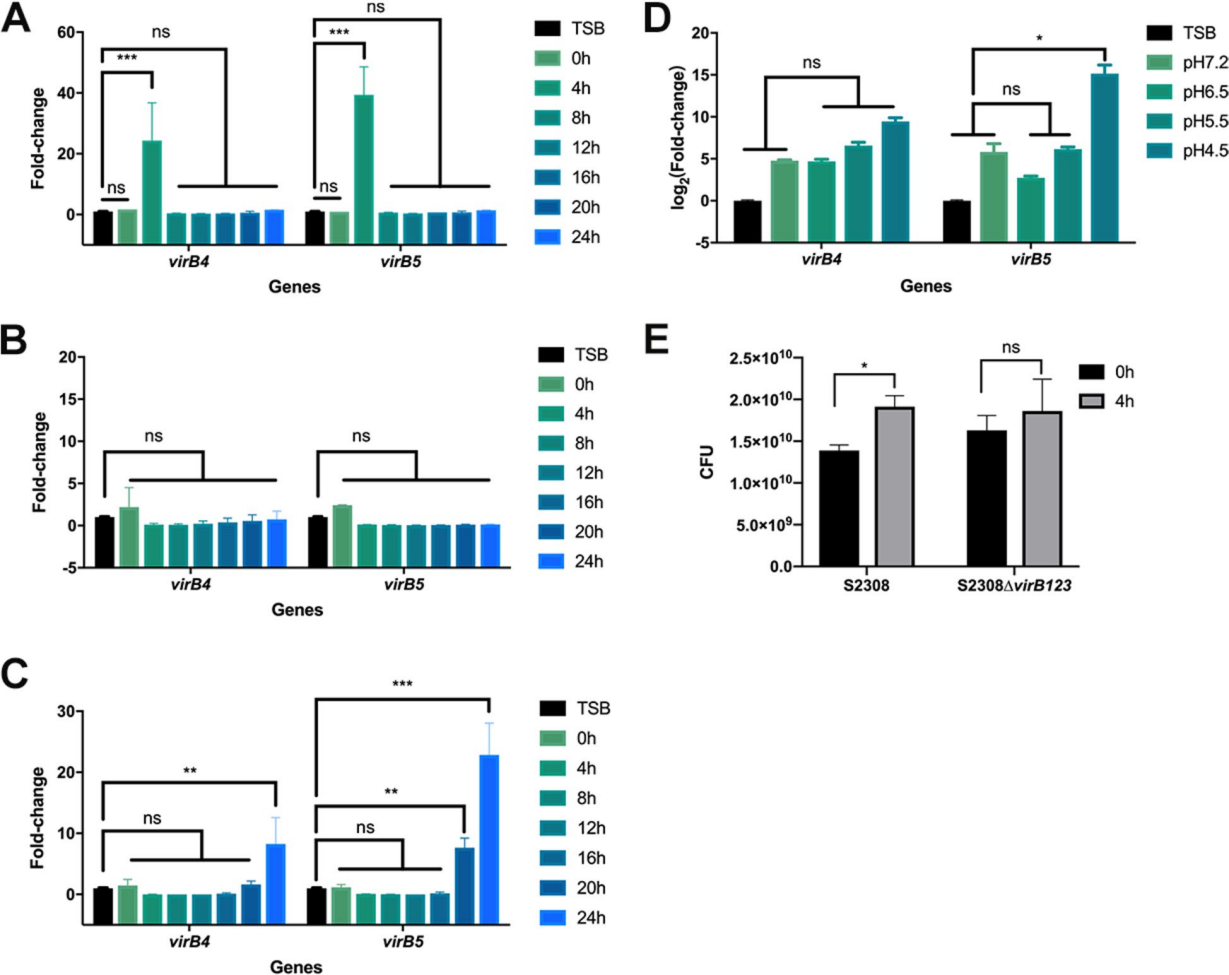
**Screening of optimal conditions for the expression of B. abortus supernatant proteins.** The expression of *B. abortus* supernatant proteins was evaluated by detecting the transcript levels of *virB4* and *virB5.* **A** Expression induced in RPMI 1640; **B** expression induced in GW medium; **C** expression induced in defined medium. **D** Expression induced in RPMI 1640 medium at different pH values for 4 h. The transcript levels of *virB4* and *virB5* were detected by qPCR, with *gapdh* as an internal reference (*N* = 3, means ± SD, two-way ANOVA). **E** Bacterial CFUs of *B. abortus* S2308 and S2308∆*virB123* in vitro in a pH = 4.5 environment (*N* = 4, means ± SD, two-way ANOVA). *p < 0.05; **p < 0.01; ***p < 0.001; ns: nonsignificant difference.

*B. abortus* was cultured in vitro for 24 h in RPMI 1640 medium, Gerhardt and Wilson (GW) medium [12, 13], or specific defined medium [14]. To evaluate T4SS secretion, bacterial samples were collected every 4 h for RNA extraction and qPCR detection of the transcription levels of *virB4* and *virB5*, with *gapdh* as the internal reference. Compared with those in the TSB culture before induction, the transcription levels of *virB4* and *virB5* were upregulated by 24.35- and 39.40-fold, respectively, after 4 h of induction in RPMI 1640 medium (Figure 1A), but no significant difference was detected when these genes were induced in GW medium for 0–24 h (Figure 1B). When induced in specific defined medium, the transcript level of *virB4* was upregulated 8.25-fold at 24 h, and *virB5* was upregulated 7.64- and 22.85-fold at 20 h and 24 h of induction, respectively (Figure 1C). Thus, we chose RPMI 1640 medium and a 4 h incubation time for collecting bacterial culture supernatants. In addition, given that an acidic environment favours the acidification of *Brucella*-containing vacuoles (BCVs), which promote T4SS expression, facilitate *Brucella* adaptation to the host intracellular environment and survive in host cells by adopting a stealth strategy [23, 24], we sought to investigate the acidic environment that can induce increased T4SS expression. RPMI 1640 medium at different pH values of 6.5, 5.5, and 4.5 was used for the induction of *Brucella* strains for 4 h. The *virB4* and *virB5* transcript levels were highest at pH 4.5 (Figure 1D). In addition, the CFU counts of S2308 and SV123 did not decrease in RPMI 1640 medium at pH 4.5, indicating that pH 4.5 had no effect on the survival of *Brucella* (Figure 1E).

### Quantitative proteomics analysis of the differential secretory proteins of S2308 and SV123

*B. abortus* secretory proteins were induced under the optimal conditions described above, and three replicates for each strain were collected. In the screening of differential secretory proteins, we obtained 15 upregulated proteins with a fold change (FC) > 2.0-fold and a *P* value < 0.05 (*t* test or others) in S2308 compared with SV123. Using at least two of three replicates identified in the S2308 samples and all null values in the SV123 samples as criteria, 4 proteins present in S2308 but absent in SV123 were obtained (Table 1).

**Table 1:** **Information on differential supernatant proteins from** ***Brucella*** **S2308 and its SV123 mutant obtained via label-free quantitative proteomics analysis.**

Abbreviation^a^	Protein (S2308)^b^	Gene (S2308)^c^	Protein (M28)^d^	Gene (M28)^e^	Interspecific conservation^f^ (%)	Annotation^g^	S2308/SV123^h^
RS14565	WP_002967213.1	BAB_RS27780	WP_004682335.1	BM28_RS14565	99.68	2-Dehydro-3-deoxygalactonokinase	5.22
RS12570	WP_002972046.1	BAB_RS29800	WP_002972046.1	BM28_RS12570	100	Cytochrome bd-I oxidase subunit CydX	3.50
RS00505	WP_002969688.1	BAB_RS16420	WP_005970254.1	BM28_RS00505	99.26	YdcF family protein	3.41
RS00230	WP_002969660.1	BAB_RS16150	WP_005970293.1	BM28_RS00230	99.94	translocation/assembly module TamB	3.00
RS03895	WP_002963941.1	BAB_RS19875	WP_004686484.1	BM28_RS03895	99.58	NADH-quinone oxidoreductase subunit NuoE	2.96
RS08185	WP_002964816.1	BAB_RS24235	WP_004686300.1	BM28_RS08185	99.75	Phosphoglycerate kinase	2.77
RS03635	WP_002963891.1	BAB_RS19630	WP_004685557.1	BM28_RS03635	99.5	Hypothetical protein	2.64
RS08115	WP_002964802.1	BAB_RS24165	WP_002964802.1	BM28_RS08115	100	Peptidoglycan-binding protein	2.63
RS02205	WP_002963604.1	BAB_RS18180	WP_002963604.1	BM28_RS02205	100	CvpA family protein	2.58
RS12560	WP_002967308.1	BAB_RS29810	WP_002967308.1	BM28_RS12560	100	Cytochrome ubiquinol oxidase subunit I	2.56
RS12665	WP_002966097.1	BAB_RS29695	WP_002966097.1	BM28_RS12665	100	ABC transporter	2.18
RS10585	WP_006077544.1	BAB_RS26665	WP_002966514.1	BM28_RS10585	99.58	P-type DNA transfer protein VirB5	+ ∞
RS15060	WP_002968608.1	BAB_RS31060	WP_002968608.1	BM28_RS15060	100	Complex I NDUFA9 subunit family protein	+ ∞
RS10635	WP_002966504.1	BAB_RS26715	WP_004681235.1	BM28_RS10635	99.7	SPFH/Band 7/PHB domain protein	+ ∞
RS01925	WP_002963543.1	BAB_RS17890	WP_002963543.1	BM28_RS01925	100	F0F1 ATP synthase subunit A	+ ∞

^a^The proteins are abbreviated as the last seven characters of the *Brucella* M28 genes.

^b^Differential supernatant proteins from *Brucella* S2308 and its SV123 mutant as determined by label-free quantitative proteomics analysis.

^c^Coding genes of *Brucella* S2308.

^d^Homologous proteins in *Brucella* M28.

^e^Homologous genes in *Brucella* M28.

^f^Conservation rates of the *Brucella* M28 protein compared with the *Brucella* S2308 protein.

^g^Predicted function of *Brucella* M28 proteins.

^h^Fold change in *Brucella* S2308/mutant SV123.

WP_002967213.1 was annotated as 2-dehydro-3-deoxygalactose kinase and was enriched 5.22-fold. Its homologous protein in *B. melitensis* M28 is WP_004682335.1, encoded by the gene *BM28_RS14565*, thus abbreviated as RS14565 in this study. The other 14 differentially secreted proteins are also abbreviated and summarized in Table 1.

### Translocation of *Brucella* putative effector proteins

**Figure 2:**
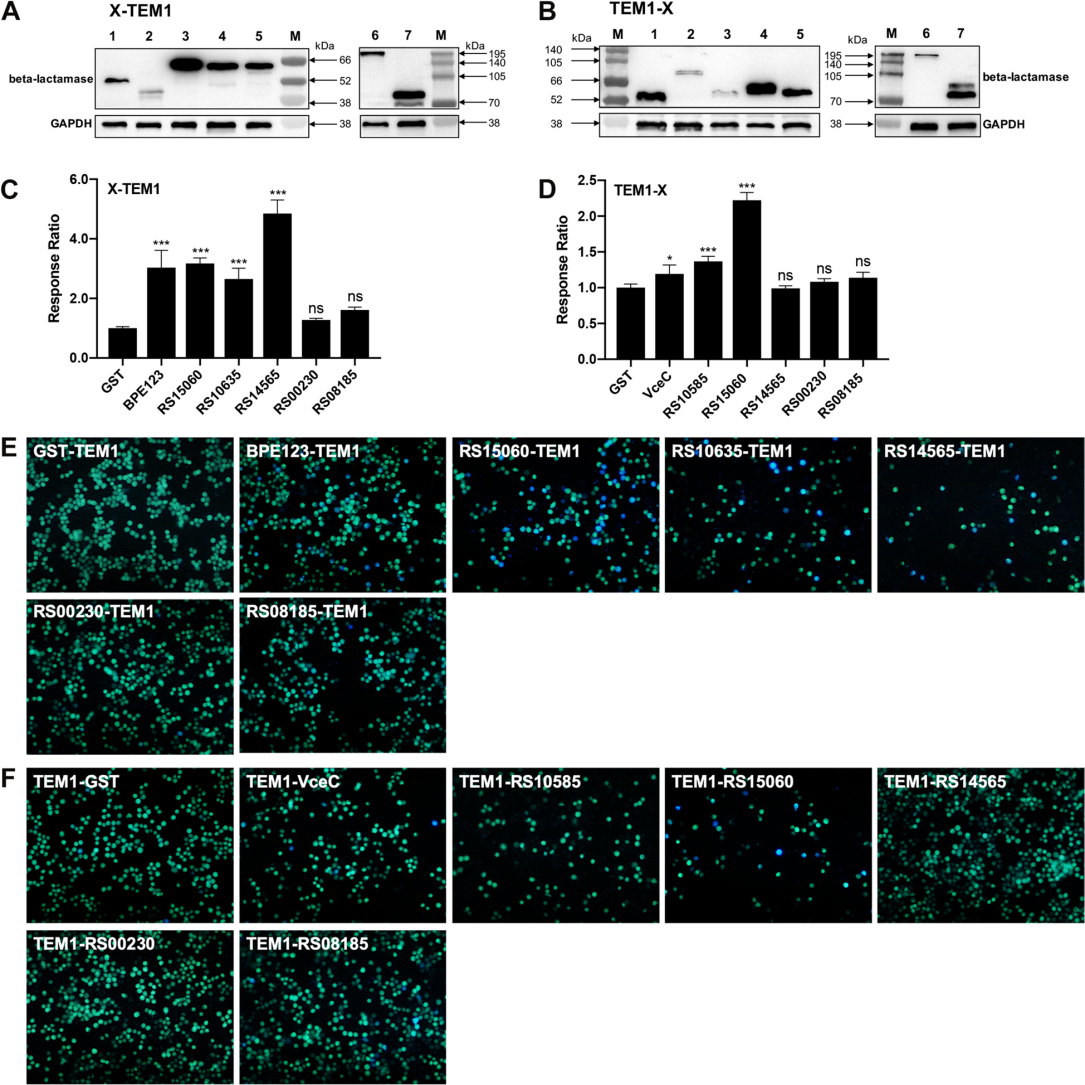
**Translocation of Brucella putative effector proteins.** **A**, **B** Western blot analysis of the expression of TEM1-tagged proteins. Bacterial lysates from the *B. melitensis* M5 strain expressing the TEM1-fusion protein were resolved by SDS‒PAGE, followed by western blot analysis with an anti-β-lactamase antibody as the primary antibody. *Brucella* GAPDH was used as a loading reference. **A** Expression of C-terminally TEM1-tagged *Brucella* proteins. Lane M: prestained protein marker VII (8–195 kDa); Lane 1: negative control protein GST-TEM1 (55.34 kDa); Lane 2: positive control protein BPE123-TEM1 (45.86 kDa); Lane 3: RS15060-TEM1 (64.99 kDa); Lane 4: RS10635-TEM1 (64.83 kDa); Lane 5: RS14565-TEM1 (61.92 kDa); Lane 6: RS00230-TEM1 (192.73 kDa); Lane 7: RS08185-TEM1 (70.97 kDa). **B** Expression of N-terminally TEM1-tagged *Brucella* proteins. Lane M: prestained protein marker VII (8–195 kDa); Lane 1: negative control protein TEM1-GST (55.47 kDa); Lane 2: positive control protein TEM1-VceC (74.21 kDa); Lane 3: TEM1-RS10585 (56.12 kDa); Lane 4: TEM1-RS15060 (65.12 kDa); Lane 5: TEM1-RS14565 (62.05 kDa); Lane 6: TEM1-RS00230 (192.84 kDa); Lane 7: TEM1-RS08185 (71.10 kDa). **C**, **E** Quantification of the translocation of C-terminally TEM1-tagged *Brucella* proteins; **D**, **F** quantification of the translocation of N-terminally TEM1-tagged *Brucella* proteins. The cytosolic translocation of β-lactamase by the *B. melitensis* M5 strain expressing different TEM1 fusion proteins was assessed via fluorescence plate-reader detection and fluorescence microscopy in RAW 264.7 cells at 5 hpi. GST-TEM1 and TEM1-GST were used as negative controls (green fluorescence), and BPE123-TEM1 and TEM1-VceC were used as positive controls (blue fluorescence). **C**, **D** The response ratios of three independent wells were calculated, and all the data were normalized so that the negative control wells had a mean value of 1.0 (*N* = 3, means ± SD, one-way ANOVA). (E–F) Representative fluorescence micrographs from individual assay wells of control proteins (GST, VceC and BPE123) and selected TEM1 fusion proteins tagged at either the C-terminus (**E**) or N-terminus (**F**). *p < 0.05; **p < 0.01; ***p < 0.001; ns: nonsignificant difference.

Among the 15 differential proteins obtained by proteomics, six proteins (RS15060, RS10635, RS14565, RS00230, RS08185 and RS10585) were selected for subsequent validation on the basis of their high enrichment (RS14565, RS00230, and RS08185) or their eukaryotic structural domains (RS15060 and RS10635). Bioinformatic analysis revealed that RS15060 is similar to eukaryotic mitochondrial NADH dehydrogenase (ubiquinone) 1 alpha subcomplex subunit 9, and the band 7 domain in RS10635 is a slipin or stomatin-like domain conserved from protozoa to mammals. RS10585 (VirB5), a component of the T4SS apparatus, was also selected for further identification. The TEM1 β-lactamase protein translocation reporter assay was used to detect whether the putative effector proteins obtained via quantitative proteomics were translocated into host cells during infection. First, given that the position of the TEM1 tag at either the C- or N-terminus of the protein can influence its translocation [19], plasmids containing translational fusions of the TEM1 protein with the C- or N-terminus of each putative effector protein were created in pT10 and transformed into the *B. melitensis* M5 strain (Additional file 2). The expression of translational fusions was detected by western blot analysis with an anti-β-lactamase antibody. The fusion proteins are shown in Figures 2A and B. Second, to assess the translocation of these TEM1 fusion proteins, RAW 264.7 cells were infected with M5 strains expressing the TEM1 fusion protein at an MOI of 1 000:1 for 5 h, after which fluorescence microscopy was performed. The TEM1-GST and GST-TEM1 fusion proteins were employed as negative translocation controls, whereas the TEM1-VceC fusion and BPE123-TEM1 fusion proteins were utilized as positive controls. Combining the blue‒green fluorescence response ratio (response ratio) obtained from the microplate reader (Figures 2C, D) and the fluorescence images taken under a fluorescence microscope (Figures 2E, F), we found that when the TEM tag was at the C-terminal end, the response ratios of RS15060, RS10635, and RS14565 increased compared with those of the GST-TEM1 (Figure 2C), and RAW 264.7 cells infected with these fusion proteins presented blue fluorescence (Figure 2E), indicating that RS15060‒TEM1, RS10635‒TEM1, and RS14565‒TEM1 could be translocated to host cells; when the TEM1 tag was at the N-terminal end, the response ratio was increased in TEM1‒RS10585 and TEM1‒RS15060 compared with that in TEM1‒GST (Figure 2D), and RAW 264.7 cells infected with TEM1‒RS15060 presented blue fluorescence under a fluorescence microscope (Figure 2F), indicating that TEM1‒RS10585 and TEM1‒RS15060 can be localized to host cells. Taken together, the results of the quantitative proteomics analysis revealed that 4 Brucella proteins were translocated into cells during infection, in which RS15060 can be translocated with the TEM1 tag at either the C- or N-terminus of the protein.

### Translocation of *Brucella* effectors reliant on the VirB T4SS

We investigated the translocation of the discovered *Brucella* effector proteins in a VirB-deficient strain to ascertain whether their translocation was VirB T4SS dependent. Thus, RS15060-TEM1, RS10635-TEM1, RS14565-TEM1, TEM1-RS15060, and TEM1-RS10585 were chosen to construct fusion proteins in the VirB-deficient strain M5∆*virB123*. The TEM1-GST and GST-TEM1 fusion proteins were expressed in M5∆*virB123* as negative translocation controls, whereas the TEM1-VceC fusion and BPE123-TEM1 fusion proteins were expressed in M5∆*virB123* as positive controls.

**Figure 3:**
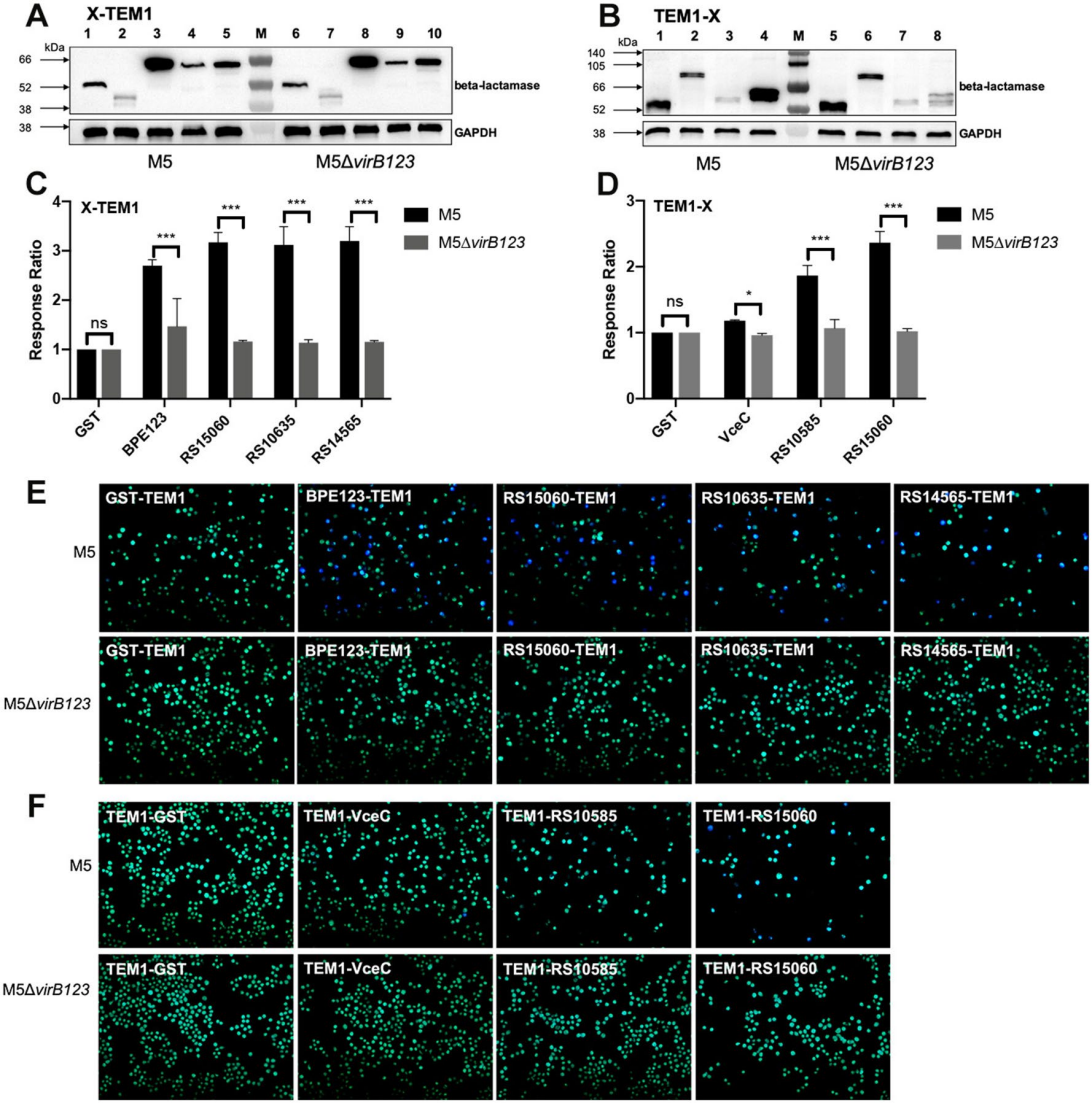
**T4SS-dependent translocation of potential Brucella effectors.** **A**, **B** Western blot analysis of the expression of TEM1-tagged proteins. Bacterial lysates from the *B. melitensis* M5 strain and M5∆*virB123* strain expressing TEM1-fusion proteins were analysed via SDS‒PAGE followed by western blot analysis, with an anti-β-lactamase antibody used as the primary antibody. *Brucella* GAPDH was used as a loading reference. **A** Expression of C-terminally TEM1-tagged *Brucella* proteins. Lane M: prestained protein marker VII (8–195 kDa). Lanes 1–5: *B. melitensis* M5 strains expressing TEM1-fusion proteins; Lanes 6–10: *B. melitensis* M5∆*virB123* strains expressing TEM1-fusion proteins. Lanes 1 and 6: negative control protein GST-TEM1 (55.34 kDa); Lanes 2 and 7: positive control protein BPE123-TEM1 (45.86 kDa); Lanes 3 and 8: RS15060-TEM1 (64.99 kDa); Lanes 4 and 9: RS10635-TEM1 (64.83 kDa); Lanes 5 and 10: RS14565-TEM1 (61.92 kDa). **B** Expression of N-terminally TEM1-tagged *Brucella* proteins. Lane M: prestained protein marker VII (8–195 kDa). Lanes 1–4: *B. melitensis* M5 strains expressing the TEM1-fusion protein; Lanes 5–8: *B. melitensis* M5∆*virB123* strains expressing the TEM1-fusion protein. Lanes 1 and 5: TEM1-GST, a negative control protein (55.47 kDa); Lanes 2 and 6: TEM1-VceC, a positive control protein expressing the full length of VceC (74.21 kDa); Lanes 3 and 7: TEM1-RS10585 (56.12 kDa); Lanes 4 and 8: TEM1-RS15060 (65.12 kDa). **C**, **E** Quantification of the translocation of C-terminally TEM1-tagged *Brucella* proteins; **D**, **F** quantification of the translocation of N-terminally TEM1-tagged *Brucella* proteins. The cytosolic translocation of β-lactamases by the M5 strain and M5∆*virB123* strain expressing different TEM1 fusion proteins was assessed by fluorescence plate-reader detection and fluorescence microscopy at 5 hpi in RAW 264.7 cells. GST-TEM1 and TEM1-GST were used as negative controls (green fluorescence), and BPE123-TEM1 and TEM1-VceC served as positive controls (blue fluorescence). **C**, **D** The response ratios of three independent wells were calculated, and all the data were normalized so that the negative control wells had a mean value of 1.0 (*N* = 3, means ± SD, two-way ANOVA). **E**, **F** Representative fluorescence micrographs of control proteins (GST, VceC and BPE123) and fusion proteins with TEM1 tags located at the C-terminus (**E**) or N-terminus (**F**) in strains M5 and M5∆*virB123*, respectively, are shown. **p < 0.01; ***p < 0.001; ns: nonsignificant difference.

Western blot analysis revealed that RS15060-TEM1, RS10635-TEM1, RS14565-TEM1, TEM1-RS10585, and TEM1-RS15060 were expressed in both M5 and M5∆*virB123* (Figures 3A, B). RAW 264.7 cells were infected with TEM1 fusion-expressing M5 or M5∆*virB123* at an MOI of 1000:1 for 5 h and then processed for fluorescence microscopy examination. In the absence of the T4SS, the response ratios of the RS14565, RS10585, RS15060, and RS10635 fusion proteins were significantly lower than those of the negative controls GST-TEM and TEM-GST in the infected RAW 264.7 cells (Figures 3C–F). Thus, the localization of the RS14565, RS10585, RS15060, and RS10635 proteins was demonstrated to be dependent on the T4SS.

### RS15060 and RS10635 are T4SS effector proteins

**Figure 4:**
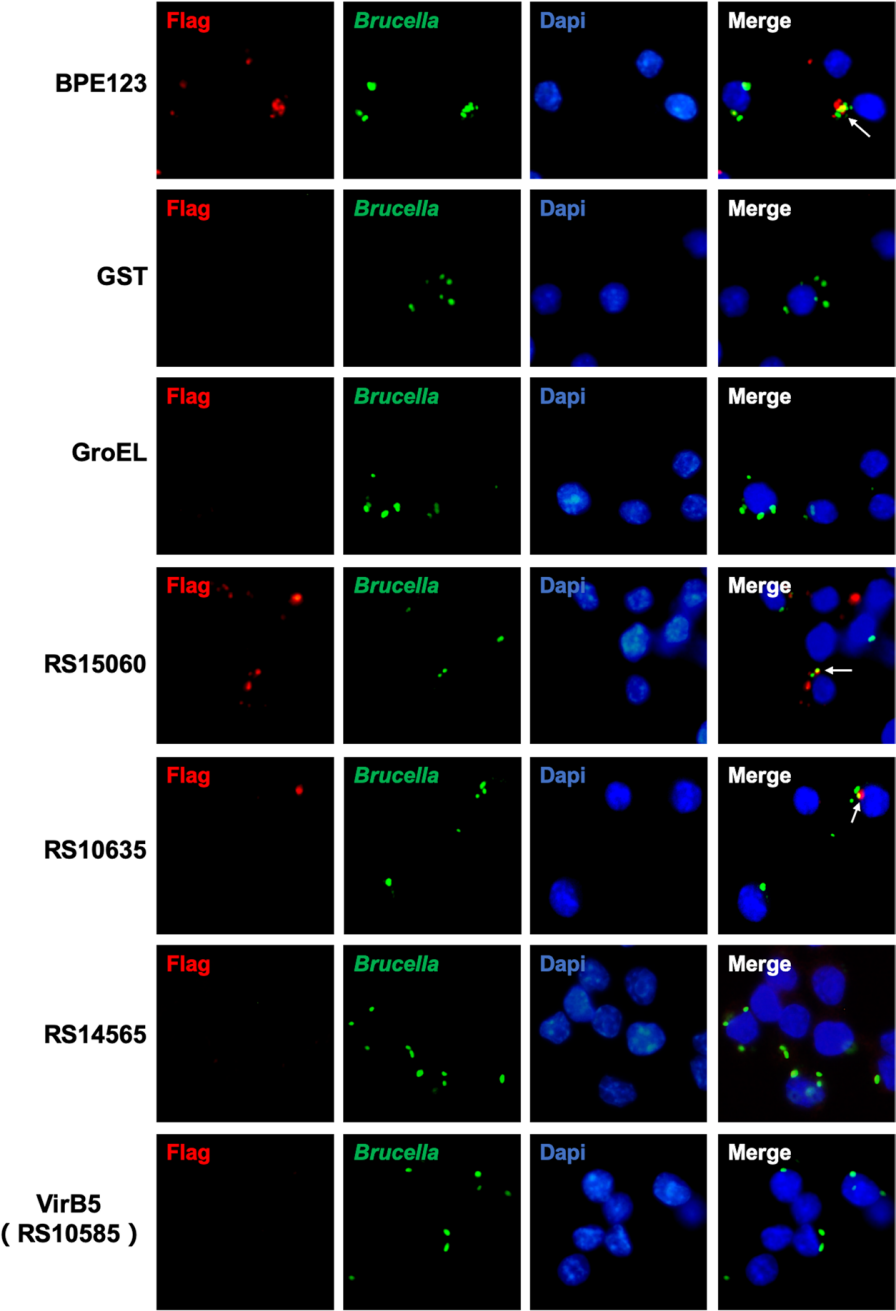
**Translocation of putative effectors identified by IFA.** Representative confocal micrographs of RAW 264.7 cells infected with *B. melitensis* M5 expressing the putative protein 3 × FLAG (MOI 100:1). M5 (BPE123-3 × FLAG) was used as a positive control, and M5 (GST-3 × FLAG) and M5 (GroEL-3 × FLAG) were used as negative controls. At 5 hpi, the cells were fixed and processed for immunostaining. FLAG staining is indicative of protein translocation across the bacterial cell envelope. The white arrows indicate the colocalization of FLAG-positive staining and anti-*Brucella* antibody staining.

To further confirm the results from the TEM1 reporter analysis system, RAW 264.7 cells were infected with *B. melitensis* M5 expressing a putative effector with 3 × FLAG (Additional file 2) and then subjected to immunofluorescence staining of FLAG epitopes (red) and bacteria (green). Since monoclonal anti-FLAG M2 antibodies remain impermeable to bacteria, stained FLAG proteins were identified as bacterial secretion proteins. GST (glutathione S-transferase) and GroEL (cytoplasmic chaperonin) are intrabacterial proteins that serve as negative controls, demonstrating that the anti-FLAG M2 antibody does not detect proteins inside the bacteria and that FLAG-positive proteins are secreted by the bacteria rather than leaked during the fixation or permeabilization process. As shown in Figure 4, RS15060 and RS10635 were detected with FLAG epitopes and showed similar red fluorescent localization to the reported effector protein BPE123, whereas no FLAG localization was observed for RS14535 and RS10585 (VirB5), confirming that RS15060 and RS10635 are T4SS effector proteins.

### RS15060 contributes to *Brucella* virulence

**Figure 5:**
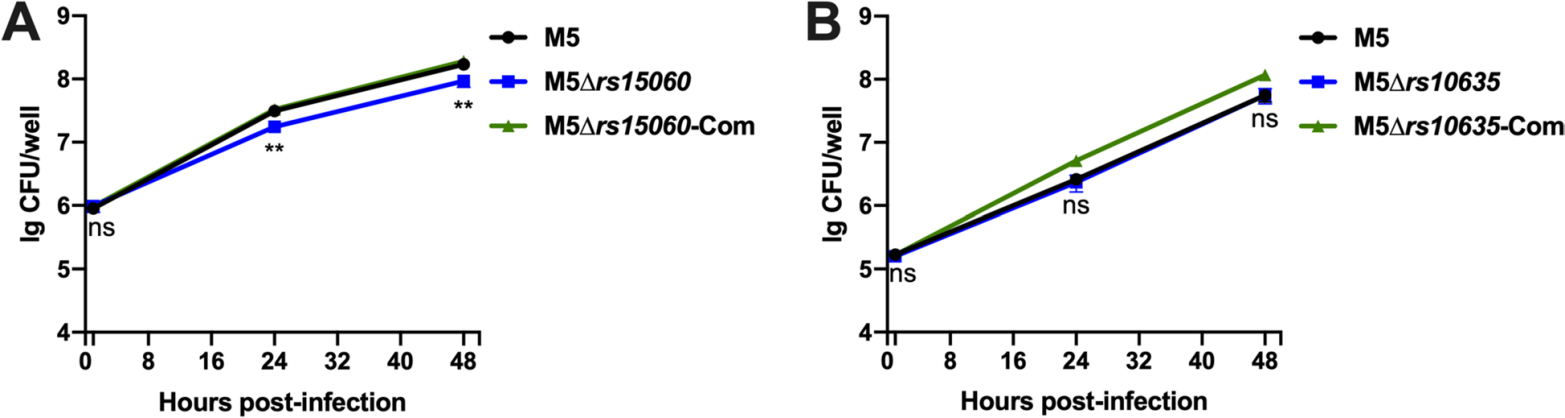
**Intercellular survival of RS15060 and RS10635.** The intracellular growth of *B. melitensis* M5 and its derived mutants was determined in RAW 264.7 cells. The cells were infected at an MOI of 200:1, and the intracellular CFUs were counted at designated points post infection. **A** *B. melitensis* M5 and its RS15060 variants; **B** *B. melitensis* M5 and its RS10635 variants. The data shown are the means ± SDs from a representative experiment performed in triplicate (*n* = 3, means ± SD, unpaired Student’s *t* test). The intracellular CFUs of *B. melitensis* M5 and its derived mutants M5∆*rs15060* and M5∆*rs10635* were statistically analysed. **p < 0.01; ns: nonsignificant difference.

Since RS15060 and RS10635 were shown to be effectors of the *Brucella* T4SS, a virulence factor related to *Brucella* survival and replication [6], we artificially knocked out the genes encoding the putative effectors and constructed complemented strains of the corresponding genes to verify whether the putative effectors are related to the virulence of *Brucella*. By counting bacterial survival in the cells, we detected no significant change in the replication ability of the bacteria between the M5 strain and the M5∆*rs10635* mutant, whereas deletion of *rs15060* impaired bacterial replication, suggesting that RS15060 affected *Brucella* intracellular survival (Figures 5A, B).

**Figure 6:**
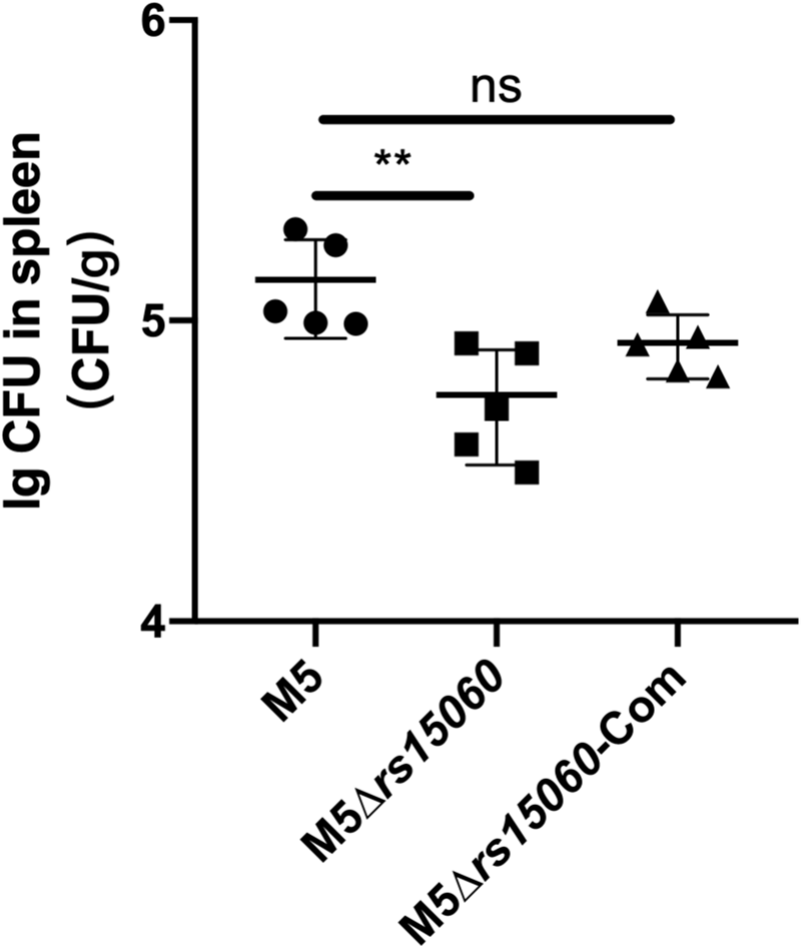
**Determination of Brucella RS15060 as a virulence factor in a mouse model.** The bacterial loads in the spleens of the mice infected with *B. melitensis* M5, the mutant M5∆*rs15060* and the complemented strain M5∆*rs15060*-Com were measured at 28 days post-infection. The bacterial CFUs of the strains were statistically analysed to determine the effect of RS15060 on *Brucella* pathogenesis (*N* = 5, means ± SD, one-way ANOVA). **p < 0.01; ns: nonsignificant difference.

To further explore whether RS15060 affects bacterial virulence in vivo, mice were infected with the M5 strain, its *rs15060* deletion mutant M5∆*rs15060*, and the complemented strain M5∆*rs15060*-Com, and the spleen bacterial load was statistically analysed at 28 days post infection. Compared with the M5 strain, the M5∆*rs15060* mutant displayed a significant replication defect in infected mice, whereas M5∆*rs15060*-Com recovered the bacterial loads in the spleen, suggesting that the deletion of *rs15060* reduced the ability of *Brucella* to proliferate and establish a chronic infection (Figure 6).

## Discussion

T4SS is a transmembrane multiprotein complex that can secrete protein and DNA molecules into the environment or directly into any prokaryotic or eukaryotic cell type. Several Gram-negative bacterial pathogens, such as *Brucella* spp., *Legionella pneumophila*, *Bartonella* spp., *Helicobacter pylori*, *Bordetella pertussis*, and *Rickettsia prowazekii*, utilize T4SS to transport effector proteins into host cells to accomplish their pathogenic processes [25]. The currently reported T4SS effectors were obtained by in silico screening and bioinformatics analysis with several indicators, such as homology with other bacterial T4SS effectors, the presence of eukaryotic-like structures, features similar to those reported *for Brucella* T4SS effectors, the presence of protein‒protein interaction domains, and the amino acid GC content [19, 21]. Proteins that meet a certain index are then confirmed by the CyaA reporter assay or the TEM1 reporter assay. This approach screens out several *Brucella* effectors but requires much work for subsequent validation. RicA has been discovered from secreted proteins in vitro [14]; therefore, new effectors may be discovered by triggering in vitro secretion. To identify additional T4SS effectors associated with *Brucella* pathogenesis, we performed a comparative proteomics analysis of the proteins secreted from *B. abortus* 2308 and its T4SS-deficient SV123 mutant and identified several novel T4SS effectors. The screening process was made more straightforward by obtaining differentially secreted proteins from wild-type strains and their T4SS-deficient strains; subsequently, via TEM1 reporter analysis and FLAG epitope staining, we identified two novel T4SS effectors. However, VceC, BPE043, and BspE, three reported T4SS effectors, were detected in the database of differentially expressed proteins in this study (Additional file 3) but failed to meet both the criteria of FC > 2.0-fold and *P* value < 0.05; thus, they are not identified as differentially secreted proteins in this study, suggesting that our comparative proteomics needs further optimization.

For subsequent validation studies, we selected the *B. melitensis* strain M5 to construct derived strains for validation of the 15 differentially expressed proteins. The reasons for using *B. melitensis* M5 for subsequent validation could be summarized as follows: (1) the genomes of *B. melitensis* and *B. abortus* share over 99% similarity (Table 1); (2) M5 is an attenuated strain that poses relatively low biosafety risks in the laboratory; and (3) M5 shows similar intracellular survival as 2308 in RAW264.1 cells at 48 hpi [17]. (4) M5 infection results in a bacterial load of up to 10^5^ CFU per gram of spleen in infected mice; thus, M5 infection could be used for virulence tests. (5) The *B. abortus* vaccine strain A19 cannot establish a useful cellular infection or a mouse infection [26]. With respect to the reported effectors, a TEM1 reporter assay was used to verify whether the potential proteins obtained via proteomics analysis were secreted into host cells [7, 8, 10]. In this study, four proteins, RS14565, RS15060, RS10635, and RS10585, were shown to localize to host cells in a T4SS-dependent manner via TEM1 analysis. However, RS10585 is a component (VirB5) of the T4SS, which is positive for TEM1, possibly because VirB5 is located on the T-shaped structure of the T4SS apparatus and extends out of the outer membrane [27]. This led us to consider whether the TEM1 reporter assay is effective in distinguishing proteins that are external to the bacteria or those attached to the bacterial membrane. Therefore, further FLAG epitope staining experiments were performed to verify whether proteins identified by TEM1 for translocation to the cell are secreted effectors. Finally, we identified RS15060 and RS10635 as novel *Brucella* T4SS effectors.

RS15060 is annotated as a complex I NDUFA9 subunit family protein, similar to eukaryotic mitochondrial NADH dehydrogenase [ubiquinone] 1 alpha subcomplex subunit 9 in *B. melitensis* M28, which is an accessory subunit for proper complex I assembly [28, 29]. Mitochondria act as the energy factory of the cell to produce ATP, which is involved in several aspects of cellular metabolism, signalling, and the immune response. The translocation of *Brucella* to the rough endoplasmic reticulum in host cells results in mitochondrial-like functions [30], suggesting that RS15060 may be able to provide energy for *Brucella* translocation or alter the metabolic state of the host cell by affecting mitochondrial function, thus providing a favourable environment for *Brucella* survival. Given that the expression of mitochondria-related genes involved in protein synthesis and transport, electron transfer, and small molecule translocation was significantly downregulated at 4 hpi, preventing the release of cytochrome c and the production of reactive oxygen species in the mitochondria, which in turn prevented the activation of the caspase cascade and inhibited macrophage apoptosis [31], RS15060 may have exerted some kind of mitochondrial function to inhibit the initiation of the apoptotic program of the host cells, thus facilitating the survival and replication of *Brucella* in the host cells. Moreover, RS15060 belongs to the subgroup of extended SDR-like proteins that are atypical SDRs. Atypical SDRs lack the catalytic residues characteristic of SDRs, and their glycine-rich NAD(P)-binding motif is often different from the forms normally observed in classical SDRs. However, they have the YXXXK active site motif and a glycine-rich NAD(P)-binding motif similar to the typical SDR, GXXGXXG. RS15060’s eukaryotic mitochondrial features and Rossmann-fold NAD(P)H/NAD(P)(+) binding (NADB) domain indicate its potential function in oxidoreductase activity, which may be related to its transmembrane transport action [32]. The YXXXK active site and the GXXGXXG site may be crucial in this process. In addition, RS15060 has an isomerase structure and belongs to the family of NAD-dependent isomerase/dehydrogenases, a family of proteins that utilize NAD as a cofactor for a variety of chemical reactions. NAD-dependent epimerase/dehydratase plays a vital role in cell surface properties and virulence in *Pectobacterium carotovorum* [33], which implies that RS15060 affects *Brucella* virulence possibly through its NAD-dependent epimerase/dehydratase activity. The mechanism by which RS15060 functions in *Brucella* virulence needs further investigation.

In conclusion, this study identified two novel effectors secreted by the *Brucella* T4SS and determined their relationship with *Brucella* virulence, which is conducive to complementing and perfecting the roles played by T4SS in the survival and replication of *Brucella* intracellularly as well as the establishment of *Brucella* as a chronic infectious process in the host. Further studies focusing on the mechanisms of these novel effectors should reveal new insights into the pathogenesis of brucellosis and provide a theoretical basis for the development of brucellosis vaccines and the formulation of preventive and control measures.

## Supplementary Information

**Additional file 1: Primers used in this study.**

**Additional file 2: Strains and plasmids used in this study.**

**Additional file 3: Significantly different supernatant proteins of Brucella S2308 vs its SV123 mutant according to proteomics.**

**Additional file 4: Presence or absence of supernatant proteins from Brucella S2308 and its SV123 mutant, as determined by proteomics analysis.**

## Data Availability

The data supporting the conclusions of this article are included within the article. Additional data used and/or analysed during the current study are available from the corresponding author upon reasonable request.

## References

[1] Whatmore AM (2009) Current understanding of the genetic diversity of *Brucella*, an expanding genus of zoonotic pathogens. Infect Genet Evol 9:1168–1184 19628055 10.1016/j.meegid.2009.07.001

[2] Atluri VL, Xavier MN, de Jong MF, den Hartigh AB, Tsolis RM (2011) Interactions of the human pathogenic *Brucella* species with their hosts. Annu Rev Microbiol 65:523–541 21939378 10.1146/annurev-micro-090110-102905 PMC13363517

[3] Moreno E, Cloeckaert A, Moriyon I (2002) *Brucella* evolution and taxonomy. Vet Microbiol 90:209–227 12414145 10.1016/s0378-1135(02)00210-9

[4] Seleem MN, Boyle SM, Sriranganathan N (2008) *Brucella*: a pathogen without classic virulence genes. Vet Microbiol 129:1–14 18226477 10.1016/j.vetmic.2007.11.023

[5] Cascales E, Christie PJ (2003) The versatile bacterial type IV secretion systems. Nat Rev Microbiol 1:137–149 15035043 10.1038/nrmicro753 PMC3873781

[6] Xiong X, Li BW, Zhou ZX, Gu GJ, Li MJ, Liu J, Jiao HW (2021) The VirB system plays a crucial role in intracellular infection. Int J Mol Sci 22:13637 34948430 10.3390/ijms222413637 PMC8707931

[7] Ke Y, Wang Y, Li W, Chen Z (2015) Type IV secretion system of Brucella spp. and its effectors. Front Cell Infect Microbiol 5:72 26528442 10.3389/fcimb.2015.00072 PMC4602199

[8] Ma ZC, Li RR, Hu RR, Deng XY, Xu YM, Zheng W, Yi JH, Wang Y, Chen CF (2020) *Brucella abortus* BspJ is a nucleomodulin that inhibits macrophage apoptosis and promotes intracellular survival of *Brucella*. Front Microbiol 11:599205 33281799 10.3389/fmicb.2020.599205 PMC7688787

[9] Luizet JB, Raymond J, Lacerda TLS, Barbieux E, Kambarev S, Bonici M, Lembo F, Willemart K, Borg JP, Celli J, Gérard FCA, Muraille E, Gorvel JP, Salcedo S (2021) The *Brucella* effector BspL targets the ER-associated degradation (ERAD) pathway and delays bacterial egress from infected cells. Proc Natl Acad Sci USA 118:e2105324118 34353909 10.1073/pnas.2105324118 PMC8364137

[10] Louche A, Blanco A, Lacerda TLS, Cancade-Veyre L, Lionnet C, Berge C, Rolando M, Lembo F, Borg JP, Buchrieser C, Nagahama M, Gérard FCA, Gorvel JP, Gueguen-Chaignon V, Terradot L, Salcedo SP (2023) *Brucella* effectors NyxA and NyxB target SENP3 to modulate the subcellular localisation of nucleolar proteins. Nat Commun 14:102 36609656 10.1038/s41467-022-35763-8 PMC9823007

[11] Gimenez A, Del Giudice MG, Lopez PV, Guaimas F, Samano-Sanchez H, Gibson TJ, Chemes LB, Arregui CO, Ugalde JE, Czibener C (2024) *Brucella* NpeA is a secreted Type IV effector containing an N-WASP-binding short linear motif that promotes niche formation. MBio 15:e0072624 38847540 10.1128/mbio.00726-24 PMC11253601

[12] Gerhardt P (1958) The nutrition of brucellae. Bacteriol Rev 22:81–98 13546130 10.1128/br.22.2.81-98.1958 PMC180938

[13] Zuniga-Ripa A, Barbier T, Conde-Alvarez R, Martinez-Gomez E, Palacios-Chaves L, Gil-Ramirez Y, Grillo MJ, Letesson JJ, Iriarte M, Moriyon I (2014) *Brucella abortus* depends on pyruvate phosphate dikinase and malic enzyme but not on Fbp and GlpX fructose-1,6-bisphosphatases for full virulence in laboratory models. J Bacteriol 196:3045–3057 24936050 10.1128/JB.01663-14 PMC4135635

[14] de Barsy M, Jamet A, Filopon D, Nicolas C, Laloux G, Rual JF, Muller A, Twizere JC, Nkengfac B, Vandenhaute J, Hill DE, Salcedo SP, Gorvel JP, Letesson JJ, De Bolle X (2011) Identification of a *Brucella* spp. secreted effector specifically interacting with human small GTPase Rab2. Cell Microbiol 13:1044–1058 21501366 10.1111/j.1462-5822.2011.01601.x

[15] Wang F, Hu S, Gao Y, Qiao Z, Liu W, Bu Z (2011) Complete genome sequences of *Brucella melitensis* strains M28 and M5–90, with different virulence backgrounds. J Bacteriol 193:2904–2905 21478357 10.1128/JB.00357-11 PMC3133110

[16] Bao Y, Tian M, Li P, Liu J, Ding C, Yu S (2017) Characterization of *Brucella abortus* mutant strain Delta22915, a potential vaccine candidate. Vet Res 48:17 28376905 10.1186/s13567-017-0422-9 PMC5381064

[17] Yin Y, Fang T, Lian Z, Zuo D, Hu H, Zhang G, Ding C, Tian M, Yu S (2023) Erythronate utilization activates VdtR regulating its metabolism to promote *Brucella* proliferation, inducing abortion in mice. Microbiol Spectr 11:e0207423 37671873 10.1128/spectrum.02074-23 PMC10580937

[18] Choi KH, Schweizer HP (2006) mini-Tn7 insertion in bacteria with single attTn7 sites: example *Pseudomonas aeruginosa*. Nat Protoc 1:153–161 17406227 10.1038/nprot.2006.24

[19] Myeni S, Child R, Ng TW, Kupko JJ 3rd, Wehrly TD, Porcella SF, Knodler LA, Celli J (2013) *Brucella* modulates secretory trafficking via multiple type IV secretion effector proteins. PLoS Pathog 9:e1003556 23950720 10.1371/journal.ppat.1003556 PMC3738490

[20] APT-BioCloud. https://bio-cloud.aptbiotech.com/.

[21] Marchesini MI, Herrmann CK, Salcedo SP, Gorvel JP, Comerci DJ (2011) In search of *Brucella abortus* type IV secretion substrates: screening and identification of four proteins translocated into host cells through VirB system. Cell Microbiol 13:1261–1274 21707904 10.1111/j.1462-5822.2011.01618.x PMC3139020

[22] de Jong MF, Sun YH, den Hartigh AB, van Dijl JM, Tsolis RM (2008) Identification of VceA and VceC, two members of the VjbR regulon that are translocated into macrophages by the *Brucella* type IV secretion system. Mol Microbiol 70:1378–1396 19019140 10.1111/j.1365-2958.2008.06487.x PMC2993879

[23] Porte F, Liautard JP, Kohler S (1999) Early acidification of phagosomes containing *Brucella suis* is essential for intracellular survival in murine macrophages. Infect Immun 67:4041–4047 10417172 10.1128/iai.67.8.4041-4047.1999 PMC96697

[24] Boschiroli ML, Ouahrani-Bettache S, Foulongne V, Michaux-Charachon S, Bourg G, Allardet-Servent A, Cazevieille C, Liautard JP, Ramuz M, O’Callaghan D (2002) The *Brucella suis virB* operon is induced intracellularly in macrophages. Proc Natl Acad Sci USA 99:1544–1549 11830669 10.1073/pnas.032514299 PMC122227

[25] Llosa M, Roy C, Dehio C (2009) Bacterial type IV secretion systems in human disease. Mol Microbiol 73:141–151 19508287 10.1111/j.1365-2958.2009.06751.x PMC2784931

[26] Cheng Z, Li Z, Yin Y, Lian Z, Abdelgawad HA, Hu H, Guan X, Zuo D, Cai Y, Ding C, Wang S, Li T, Qi J, Tian M, Yu S (2021) Characteristics of *Brucella abortus* vaccine strain A19 reveals its potential mechanism of attenuated virulence. Vet Microbiol 254:109007 33582483 10.1016/j.vetmic.2021.109007

[27] Aly KA, Baron C (2007) The VirB5 protein localizes to the T-pilus tips in *Agrobacterium tumefaciens*. Microbiology (Reading) 153:3766–3775 17975085 10.1099/mic.0.2007/010462-0

[28] van den Bosch BJ, Gerards M, Sluiter W, Stegmann AP, Jongen EL, Hellebrekers DM, Oegema R, Lambrichs EH, Prokisch H, Danhauser K, Schoonderwoerd K, de Coo IF, Smeets HJ (2012) Defective NDUFA9 as a novel cause of neonatally fatal complex I disease. J Med Genet 49:10–15 22114105 10.1136/jmedgenet-2011-100466

[29] Baertling F, Sanchez-Caballero L, van den Brand MAM, Fung CW, Chan SH, Wong VC, Hellebrekers DME, de Coo IFM, Smeitink JAM, Rodenburg RJT, Nijtmans LGJ (2018) NDUFA9 point mutations cause a variable mitochondrial complex I assembly defect. Clin Genet 93:111–118 28671271 10.1111/cge.13089

[30] Ramirez-Romero R (1998) Is *Brucella abortus* a facultative intracellular pathogen with mitochondria-like activity? Med Hypotheses 51:41–45 9881835 10.1016/s0306-9877(98)90252-3

[31] He Y, Reichow S, Ramamoorthy S, Ding X, Lathigra R, Craig JC, Sobral BW, Schurig GG, Sriranganathan N, Boyle SM (2006) *Brucella melitensis* triggers time-dependent modulation of apoptosis and down-regulation of mitochondrion-associated gene expression in mouse macrophages. Infect Immun 74:5035–5046 16926395 10.1128/IAI.01998-05 PMC1594834

[32] Loeffen JL, Triepels RH, van den Heuvel LP, Schuelke M, Buskens CA, Smeets RJ, Trijbels JM, Smeitink JA (1998) cDNA of eight nuclear encoded subunits of NADH:ubiquinone oxidoreductase: human complex I cDNA characterization completed. Biochem Biophys Res Commun 253:415–422 9878551 10.1006/bbrc.1998.9786

[33] Islam R, Brown S, Taheri A, Dumenyo CK (2019) The gene encoding NAD-dependent epimerase/dehydratase, *wcaG*, affects cell surface properties, virulence, and extracellular enzyme production in the soft rot phytopathogen. Pectobacterium carotovorum Microorganisms 7:172 31200539 10.3390/microorganisms7060172 PMC6616942

